# Association between the DTNBP1 gene and intelligence: a case-control study in young patients with schizophrenia and related disorders and unaffected siblings

**DOI:** 10.1186/1744-9081-3-19

**Published:** 2007-04-20

**Authors:** Janneke R Zinkstok, Odette de Wilde, Therese AMJ van Amelsvoort, Michael W Tanck, Frank Baas, Don H Linszen

**Affiliations:** 1 Department of Psychiatry, Academic Medical Center of the University of Amsterdam, The Netherlands; 2 Neurogenetics Laboratory, Academic Medical Center of the University of Amsterdam, The Netherlands; 3 Department of Clinical Epidemiology, Biostatistics, and Bioinformatics, Academic Medical Center of the University of Amsterdam, The Netherlands

## Abstract

**Background:**

The dystrobrevin-binding protein 1 (DTNBP1) gene is a susceptibility gene for schizophrenia. There is growing evidence that DTNPB1 contributes to intelligence and cognition. In this study, we investigated association between single nucleotide polymorphisms (SNPs) in the DTNBP1 gene and intellectual functioning in patients with a first episode of schizophrenia or related psychotic disorder (first-episode psychosis, FEP), their healthy siblings, and unrelated controls.

**Methods:**

From all subjects IQ measurements were obtained (verbal IQ [VIQ], performance IQ [PIQ], and full scale IQ [FSIQ]). Seven SNPs in the DTNBP1 gene were genotyped using single base primer extension and analyzed by matrix-assisted laser deionization mass spectrometry (MALDI-TOF).

**Results:**

Mean VIQ, PIQ, and FSIQ scores differed significantly (p < 0.001) between patients, siblings, and controls. Using a family-based and a case-control design, several single SNPs were significantly associated with IQ scores in patients, siblings, and controls.

**Conclusion:**

Although preliminary, our results provide evidence for association between the DTNBP1 gene and intelligence in patients with FEP and their unaffected siblings. Genetic variation in the DTNBP1 gene may increase schizophrenia susceptibility by affecting intellectual functioning.

## Background

Schizophrenia is associated with cognitive impairments [1,2] as was already recognized by Kraepelin [3] who used the term "dementia praecox" to describe a chronic, deteriorating psychotic disorder characterized by progressive cognitive decline. To date, cognitive dysfunctions including deficiencies in attention, working memory, and executive functions are considered core features of the clinical schizophrenia phenotype, and are probably highly predictive for functional outcome [4,5]. No single etiological substrate for schizophrenia and its associated cognitive profile has yet been identified, but common abnormalities include reductions in neuropil and size of neurons, and structural and functional abnormalities of prefrontal and temporal regions and the hippocampal formation [6]. Also, altered glutamatergic neurotransmission has been implicated as a functional substrate for psychotic and cognitive symptoms in schizophrenia [7], in addition to the classical dopamine hypothesis [8]. Evidence from genetic linkage and association, and twin studies points to a major contribution of genetic factors to schizophrenia etiology [9]. Furthermore, since healthy family members of patients with schizophrenia show similar cognitive deficits, the cognitive profile associated with schizophrenia is probably highly heritable [10].

One of the key candidate genes to date is the dystrobrevin-binding protein 1 (DTNBP1, or dysbindin) gene. Not only have several genome scans reported linkage of chromosome 6p22–24 to schizophrenia [11-14], but also association has been reported between schizophrenia and a set of individual markers and haplotypes spanning the chromosomal region containing the DTNBP1 gene (6p22.3), a finding which has been replicated in several different populations [15-24]. Despite the large number of studies supporting DTBNP1 as a positional candidate gene for schizophrenia [25], functional variants have not yet been identified, nor have any pathophysiological mechanisms.

However, there is accumulating evidence that DTBNP1 may have a role in cognition. For example, one association study found a specific protective haplotype that was associated with higher educational achievement in patients with schizophrenia [24]. The authors propose that this protective haplotype within DTNBP1 may modify schizophrenia risk by enhancing cognition. This hypothesis is supported by a recent genome scan for intelligence using a large sample of healthy sib-pairs, that revealed putative linkage on 6p25.3-6p22.3 [26], and by a linkage scan in schizophrenia patients reporting linkage at 6p24 to a neurocognitive deficit subtype of schizophrenia [27]. In addition, post-mortem studies have shown that DTNBP1 expression is reduced in the hippocampus and prefrontal cortex of patients with schizophrenia, regions of the brain that are crucial for adequate cognitive functioning [28]. DTNBP1 could also influence schizophrenia liability and cognition by modulating glutamatergic or dopaminergic neurotransmission: DTNBP1 is located in presynaptic glutamatergic neurons and is reduced in patients with schizophrenia [29]; furthermore, down-regulation of DTNBP1 may lead to hyperactivation of midbrain dopaminergic systems [30]. Both the dopaminergic and the glutamatergic systems are key neurotransmitter systems for cognitive functioning [7,31].

Recent data from two independent groups further support a role for DTNBP1 in cognition and schizophrenia susceptibility. Burdick et al [32] recently reported association between a DTNBP1 haplotype and general cognitive ability in patients with schizophrenia and in healthy controls. Patients with schizophrenia carrying a DTNBP1 risk haplotype previously identified by Funke [21], performed worse on a neurocognitive test battery including the Wechsler Adult Intelligence Test (WAIS) – Revised than patients without the risk haplotype. Likewise, healthy controls without the risk haplotype showed higher levels of general cognitive ability than those carrying the risk haplotype. Furthermore, a recent study by Donohoe et al [33] reported that patients with schizophrenia carrying a DTNBP1 risk haplotype showed impaired spatial working memory performance in comparison to patients without the risk haplotype. In addition to cognition, DTNBP1 has recently been associated with high levels of negative symptoms in patients with schizophrenia [34,35]; these findings are relevant in this context since negative symptoms and cognitive dysfunction are highly correlated [36].

Given the evidence supporting DTNBP1 not only as a susceptibility gene for schizophrenia but also as a gene contributing to intellectual functioning, we hypothesized that variants in the DTNBP1 gene may contribute to the risk for schizophrenia by affecting cognitive abilities. To date, association between genetic variation in the DTNBP1 gene and intellectual functioning in unaffected relatives of patients with schizophrenia-spectrum disorders has not yet been studied. Therefore, we investigated association between SNPs in the DTNBP1 gene and IQ in patients with a first episode of schizophrenia or related psychotic disorder (first-episode psychosis, FEP), their unaffected siblings, and healthy controls.

## Methods

### Subjects and assessment

All patients were admitted to the Adolescent Clinic of the Academic Medical Center of the University of Amsterdam and attended a special program for adolescents with FEP. After complete description of the study to the subjects, written informed consent was obtained. The study was approved by the human subjects review board of our institution. Exclusion criteria were organic brain disease, endocrine disorder, or learning disability. Diagnosis was established according to DSM-IV [37] criteria and was based on the Mini International Neuropsychiatric Interview (MINI) [38]. At time of testing patients were clinically stable for at least four weeks (i.e. no changes in antipsychotic medication in the four weeks before testing). Additionally, healthy siblings were recruited, and unrelated healthy controls from the general population matched for age, educational level, and ethnicity. Both groups were screened for psychopathology using the MINI and were excluded when they had an Axis I disorder, organic brain disease, or learning disability. All subjects were administered the Wechsler Adult Intelligence Test – third version (WAIS III). Blood was collected from all subjects for DNA isolation. Where available, parents (of patients) were asked to provide blood for DNA isolation.

### DNA extraction and genetic analysis

Genomic DNA was extracted using a filter-based method (QIAamp DNA Mini Kit, Qiagen Ltd, United Kingdom). Genotyping was performed using single base primer extension and analyzed by matrix-assisted laser-desorption/ionization time-of-flight mass spectrometry (MALDI-TOF MS) on a Bruker III Daltonics Mass Spectrometer as described previously [39] (details available upon request). All DNA samples were genotyped in duplicate to ensure reliability.

### Choice of markers

We selected markers which showed association with schizophrenia in previous studies. We chose 6 markers (Table 1) from the original report by Straub [15] (rs2619539, rs3213207, rs1011313, rs 2619528, rs760761, and rs2619522) and 1 additional SNP first reported by Williams [24] (rs2619538).

**Table 1:** SNP information

	Inter marker distance	Nucleotide change	Minor allele frequency	SNP description Ensembl release 42	Chr 6. position March 2006 UCSC freeze
rs2619539	0	G/C	0.47	Intron 5	15728834
rs3213207	7247	A/G	0.13	Intron 4	15736081
rs1011313	5330	G/A	0.06	Intron 4	15741411
rs2619528	16397	G/A	0.30	Intron 3	15757808
rs760761	1303	C/T	0.28	Intron 3	15759111
rs2619522	2517	T/G	0.30	Intron 1	15761628
rs2619538	11560	A/T	0.49	5' flanking region	15773188

In the 3^nd ^column ("nucleotide change") the common allele is presented first. Minor allele frequency in patients is given in the 3^rd ^column.

SNP: Single Nucleotide Polymorphism. UCSC: University of California Santa Cruz, Genome Bioinformatics,

### Statistical analysis

#### 1. Demographics statistics

Differences in age and educational level between patients, siblings and controls were analyzed using analysis of variance (ANOVA), between-group differences in gender were analyzed using χ^2 ^tests. Differences in verbal IQ (VIQ), performance IQ (PIQ), and full scale IQ (FSIQ) scores between patients, siblings and controls were analyzed using ANOVA adjusting for age, gender, and educational level. The generalized estimating equations (GEE) method [40] was used to correct for correlations due to family relations. We used SAS version 9 (SAS Institute Inc., Cary NC, USA); level of statistical significance was defined as *p *<0.05.

#### 2. Association analysis with single SNPs and multiple SNP combinations in cases, siblings, and unrelated controls

Differences in allele frequencies between patients, siblings, and controls were assessed using χ^2 ^tests. We assessed the effect of single SNPs in the DTNBP1 gene on VIQ, PIQ, and FSIQ scores in patients with FEP, healthy siblings, and unrelated controls using ANOVA adjusting for age, gender, ethnicity, and educational level. The GEE method was used to correct for possible correlations between patients and siblings due to family relations. We added the rare homozygotes to the heterozygotes, thus analyzing 2 genotype groups instead of 3. Additionally, association between VIQ, PIQ, and FSIQ, and multiple SNP combinations was analyzed using backward stepwise regression analysis as described previously, with all 7 polymorphisms in the initial model together with the (fixed) covariates age, gender, ethnicity, and educational level. Level of statistical significance was defined as *p *<0.05.

#### 3. Quantitative pedigree disequilibrium test (QPDT)

In addition, we used a family-based analysis including genotype data from patients, unaffected siblings, and parents, to test for association between PIQ, VIQ, and FSIQ scores and DTNBP1 polymorphisms. In total, 52 pedigrees were included in this analysis. All 52 pedigrees included 1 proband, 26 pedigrees included 1 or more unaffected siblings; in 26 pedigrees no unaffected sibling was available. From 33/52 patients (63%) DNA from both parents was available; from 19/52 patients (37%) we had DNA from only one parent. Linkage disequilibrium (LD) between SNPs was calculated in Haploview version 3.32  with standard transmission disequilibrium test (TDT) settings and expressed as D' and r^2 ^(see Table 2). We used the quantitative pedigree disequilibrium test (QPDT) [41] implemented in UNPHASED [42].

**Table 2:** Pairwise LD in DTNBP1 polymorphisms

	rs2619539	rs3213207	rs1011313	rs2619528	rs760761	rs2619522	rs2619538
rs2619539	-	0.156	0.111	0.0	0.0	0.001	0.055
rs3213207	1.0	-	0.014	0.334	0.403	0.34	0.076
rs1011313	0.916	1.0	-	0.037	0.033	0.036	0.081
rs2619528	0.024	0.955	1.0	-	0.838	0.737	0.128
rs760761	0.015	1	1.0	0.96	-	0.854	0.166
rs2619522	0.04	0.955	1.0	0.867	0.96	-	0.182
rs2619538	0.242	0.823	0.825	0.648	0.772	0.778	-

D' values and r^2 ^values for all combinations of the 7 SNPs included in this study are calculated in Haploview version 3.32

with standard TDT settings.

Above diagonal r^2^-values are shown, below diagonal D'- values are shown.

Rare marker combinations with frequencies below 0.05 were excluded from the analysis. Global p-values were obtained for single SNPs and for 2-, 3- and 4- marker combinations in a sliding window.

## Results

### 1. Demographic data

**Figure 1:**
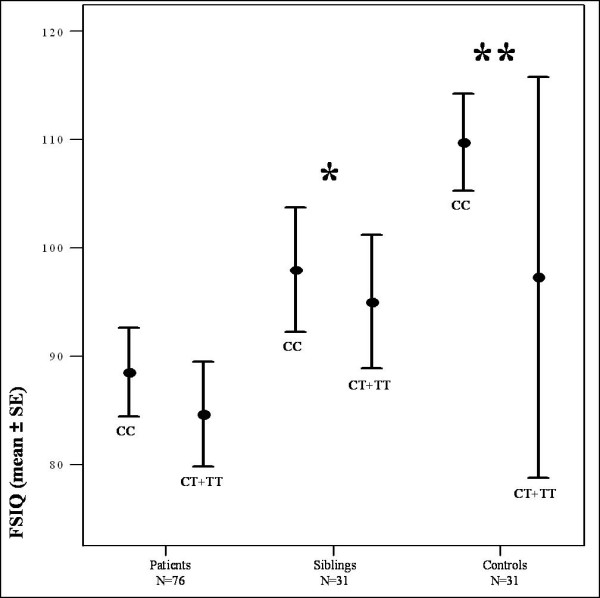
**Mean FSIQ in patients, siblings and controls**. Distribution of FSIQ by genotype within each subject group for the most consistently associated SNP (rs760761) is shown. In the whole group, FSIQ scores in patients with FEP (mean = 86.7, SEM = 1.58) were significantly lower than IQ scores in siblings (mean = 96.7, SEM = 2.08) and controls (mean = 107.0, SEM = 2.79) (ANOVA adjusting for age, gender, and educational level, p < 0.001). Post-hoc analyses showed that siblings had significantly higher IQ scores than patients (* *p *= 0.001) and significantly lower IQ scores than controls (** *p *= 0.005).

We included 76 patients with FEP, 31 healthy siblings, and 31 unrelated control subjects (see Table 3). Ethnicity counts are shown in Table 4. The patients met DSM-IV criteria for schizophrenia (N = 57; 75%), schizoaffective disorder (N = 13; 17%), brief psychotic disorder (N = 3; 4%), and psychotic disorder not otherwise specified (N = 3; 4%). Mean duration of psychosis was 20 months (SD = 15.06). Patients were receiving antipsychotic medication (80.2%), mostly atypical antipsychotics. Mean dose of antipsychotic medication in chlorpromazine equivalents was 291.06 (SD = 266.70). Six out of 76 patients (8%) were also diagnosed with mild to moderate depressive disorder and used an SSRI. None of the healthy siblings and none of the unrelated controls used any psychotropic medication. Means and numbers for age, educational level, gender, VIQ, PIQ, and FSIQ for cases, siblings and controls are presented in Table 3. All data were normally distributed. There was no difference between patients receiving typical and atypical neuroleptic treatment on IQ scores. We found no significant correlation between chlorpromazine equivalents and FSIQ (Pearson's Rho=-0.13; *p *= 0.3). Also, educational level did not differ significantly between patients, siblings and controls (*p *= 0.11). The number of males and females differed significantly between groups (*p *<0.01). Mean VIQ, PIQ, and FSIQ scores differed significantly (*p *<0.001) between patients, siblings, and controls (Figure 1). IQ scores in patients (mean VIQ = 89.2 ± 14.3, mean PIQ = 85.7 ± 13.0, mean FSIQ = 86.7 ± 13.8) were significantly lower than IQ scores in siblings (mean VIQ = 95.7 ± 11.1, mean PIQ = 98.6 ± 11.4, mean FSIQ = 96.7 ± 11.6) and controls (mean VIQ = 105.6 ± 16.9, mean PIQ = 106.4 ± 14.4, mean FSIQ = 107.0 ± 15.5). Post-hoc analyses showed that siblings had significantly lower IQ scores than controls (*p *= 0.005) and significantly higher IQ scores than patients (*p *= 0.001) (Figure 1).

**Table 3:** Subject characteristics

	***Patients N = 76***	***Siblings N = 31***	***Controls N = 31***	*Statistic*	*p-value*
Age (SD)	21.5 (2.9)	22.8 (3.8)	21.5 (3.1)	*F *= 2.27	*p *= 0.11
Gender (M/F)	66/10	13/18	23/8	χ^2 ^= 23.03	*p *<0.001
Educational level (SD)	4.45 (0.91)	4.76 (1.15)	4.83 (1.09)	*F *= 1.94	*p *= 0.15
VIQ (SD)	89.2 (14.3)	95.7 (11.1)	105.6 (16.9)	*F *= 14.68	*p *<0.001*
PIQ (SD)	85.7 (13.0)	98.6 (11.4)	106.4 (14.4)	*F *= 31.35	*p *<0.001*
FSIQ (SD)	86.7 (13.8)	96.7 (11.6)	107.0 (15.5)	*F *= 25.12	*p *<0.001*

Subject characteristics (age, gender, and educational level) and verbal, performance, and full scale IQ scores (VIQ, PIQ, and FSIQ). Between-group differences in age, educational level, and between VIQ, PIQ, and FSIQ are calculated using ANOVA. Between-group differences in gender are calculated using a χ^2 ^test. The *p*-values marked with * result from an overall ANOVA; all pairwise comparisons are significant.

**Table 4:** Overview of subjects' countries of origin

	Subject		
*Country of origin*	*Patients*	*Siblings*	*Controls*

Netherlands (white Caucasian)	48	25	22
Turkey	3	1	0
Morocco	4	0	4
Surinam or Dutch Antilles	17	3	5
Missing	4	2	0

Country of origin did not differ significantly between subject groups (Fisher's Exact = 9.44, *p *= 0.24).

### 2. Association analysis with single SNPs and marker combinations in cases, siblings, and unrelated controls

None of the SNPs deviated from Hardy-Weinberg equilibrium. Allele frequencies did not differ between probands, siblings and healthy controls (data not shown). After adjusting for age, gender, ethnicity, educational level, and family membership, 3 of the 7 SNPs examined (rs760761, rs2619522, and rs2619538) showed significant association with FSIQ scores in patients, siblings and controls (rs760761: *p *= 0.026; rs2619522: *p *= 0.025; rs2619538: *p *= 0.038; Table 5). Two SNPs showed association with VIQ, a third showed marginal significance (rs760761: *p *= 0.049; rs2619522: *p *= 0.052; rs2619538: *p *= 0.029). Furthermore, rs760761 and rs2619522 showed significant association with PIQ (*p*-values 0.038 and 0.023 respectively). Using non-parametric techniques (rank-transformation) similar *p*-values were obtained (data not shown).

**Table 5:** Mean IQ values and associations by SNP and genotype

	***Mean FSIQ (SD)***			
	Non-carriers	Carriers	Z-statistic	***Adjusted p-value***

rs 2619539	91.02 (16.02)	94.52 (15.92)	1.02	0.308
rs 3213207	94.16 (15.96)	91.30 (16.06)	-1.60	0.109
rs 1011313	93.50 (16.37)	93.29 (13.23)	1.29	0.198
rs 2619528	96.36 (15.21)	89.95 (16.30)	-1.65	0.098
rs 760761	96.79 (15.18)	88.77 (16.02)	-2.23	**0.026**
rs 2619522	96.74 (15.29)	89.23 (15.97)	-2.25	**0.025**
rs 2619538	88.52 (16.57)	95.04 (15.54)	2.08	**0.038**

Observed mean FSIQ values of the total population (patients, siblings and controls) with standard deviation (SD) are given in 'carriers' versus the 'non-carriers' in which 'carriers' represent the carriers of the low-frequency allele. *P*-values are given for the comparison of FSIQ values between genotype groups (carriers and non-carriers) using ANOVA with GEE adjusting for age, gender, ethnicity, family membership, and educational level.

When we analyzed association between IQ scores and 2-, 3-, or 4-marker combinations, none of the marker combinations showed significant association with VIQ, PIQ, or FSIQ scores (data not shown). The multiple SNP models showed that a model including only rs760761 fitted the data equally well compared to a model including all 7 SNPs, suggesting that the observed differences may be caused by a single mutation.

### 3. QPDT results

We found significant association between FSIQ scores and 4 single SNPs in the DTNBP1 gene using the QPDT (rs 2619528: *p *= 0.044; rs 760761: *p *= 0.022; rs 2619522: *p *= 0.022; rs 2619538: *p *= 0.026). None of the 2-, 3-, or 4-marker combinations showed significant association with FSIQ scores. Three single SNPs showed significant association with VIQ (rs760761: *p *= 0.027; rs2619522: *p *= 0.027; rs2619538: *p *= 0.030). The 4 SNPs associated with FSIQ were also associated with PIQ (rs2619528: *p *= 0.033; rs760761: *p *= 0.023; rs2619522: *p *= 0.024; rs2619538: *p *= 0.023). Using UNPHASED, we found LD between the 4 SNPs associated with FSIQ to be high (D' ranging from 0.7–1.0), therefore we think the SNPs are part of an LD block and do not contribute to variance in FSIQ individually.

## Discussion

In our study population we found significant association between single SNPs in the DTNBP1 gene and IQ measurements in patients with FEP, their unaffected siblings, and controls matched for age, educational level, and ethnicity. Rs760761 showed the strongest and most consistent association with IQ values in patients, unaffected siblings, and unrelated controls. Whether this association is caused by rs760761 itself or by a functional mutation in LD with this polymorphism, cannot be concluded from our data.

### Previous work on DTNBP1 and cognition

Our data are partly consistent with a recent report of association between genetic variation in DTNBP1 and measures of prefrontal brain function in healthy adults [43]. In this study the polymorphisms rs2619528 and rs760761 showed strong association with prefrontal electrophysiology as measured by event-related potentials (ERPs) both individually and as part of a haplotype. In particular the GG variant of rs2619528 and the CC variant of rs760761 were associated with impaired prefrontal brain electrophysiology. In our study population, the rs760761 CC genotype was associated with better intellectual functioning. A possible explanation for this discrepancy is that genetic variation in DTNBP1 may differentially affect electrophysiology and IQ measures. Alternatively, effects of rs760761 on prefrontal brain electrophysiology in healthy controls may differ from those in patients with schizophrenia; therefore future studies should investigate whether associations between DTNBP1 and electrophysiological measures are also present in patients with schizophrenia.

Support for a role of DTNBP1 in cognition and in schizophrenia came first from Burdick [32] who recently reported association between a DTNBP1 haplotype and general cognitive ability, and from Donohoe et al [33] who reported association between a DTNBP1 haplotype and spatial working memory in patients with schizophrenia. Our data are consistent with these studies although we used a different approach. The risk haplotype used by Donohoe et al [33] was previously identified in same cohort by Williams et al [24]; similarly, the risk haplotype used by Burdick et al [32,44] and by DeRosse et al [35] was previously identified in same sample by Funke et al [21]. We however tested SNPs in a hypothesis-free fashion. Also, the SNPs we typed did not include the tagging SNP (rs1018381) from the risk haplotype used in the sample of Funke et al [21]. And although we did type the three SNPs that formed the risk haplotype in the Donohoe et al [33] study, our study focused on general cognitive ability similar to Burdick et al [32], whereas Donohoe [33] investigated a specific cognitive process, namely spatial working memory. Therefore, results may be partly discrepant from previous studies due to differences in approach, cognitive measures, and genotyped SNPs.

### Biological mechanism

Our data add to the growing evidence that genetic variation in the DTNBP1 gene modifies schizophrenia susceptibility by influencing general cognitive abilities. Not only is the 6p22–24 locus implicated in cognition and neurocognitive deficit in schizophrenia by recent genome scans [26,27] and has DTNBP1 been associated with more severe negative symptoms which are highly correlated to cognitive dysfunction [34,45], but also recent neuropathological studies have revealed reduced expression of DTNBP1 in the prefrontal cortex [28] and in the hippocampus [29] in postmortem brain tissue of schizophrenia patients. Both structures are involved in cognitive functions, the prefrontal cortex merely in working memory and executive function, the hippocampus mainly in memory formation. Also, risk haplotypes in the DTNBP1 gene have been associated with reduced expression of DTNBP1 mRNA in brain tissue of healthy subjects and in patients with schizophrenia, whereas 'protective' haplotypes were associated with high DTNBP1 expression [46]. Changes in DTNBP1 expression are probably not caused by antipsychotic drug treatment [47] but instead may reflect an increased genetic vulnerability for schizophrenia. Thus, genetic variation in the DTNBP1 gene may modulate schizophrenia susceptibility by altering DTNBP1 expression in areas of the brain crucial for cognitive functions. This may occur already in an early stage, since genetic variation in DTNBP1 has been associated with poor premorbid function in children developing childhood-onset schizophrenia [48], suggesting that DTNBP1 contributes to early neurodevelopmental impairment. A possible mechanism by which dysbindin contributes to schizophrenia susceptibility, may be by modulating neurotransmitter systems in regions of the brain that are crucial for cognitive functioning and that are probably disturbed in schizophrenia: the glutamatergic system in the hippocampal formation and the dopaminergic system in the prefrontal cortex.

Recently, Talbot [29] investigated expression and localization of DTNBP1 in postmortem brain tissue of schizophrenia patients and reported evidence for a relationship between DTNBP1 and hippocampal glutamate neurotransmission: DTNBP1 expression was reduced in terminal fields of intrinsic glutamatergic connections and these reductions were related to glutamatergic alterations in intrinsic hippocampal formation connections [29]. Glutamatergic neurotransmission in the hippocampus is important for memory formation [49], therefore reductions in DTNBP1 in the hippocampus of schizophrenia patients as observed by Talbot [29] may disturb normal information processing and diminish synaptic plasticity in the hippocampus. The hippocampus has been implicated in schizophrenia pathogenesis previously, both by structural neuroimaging studies reporting reductions of hippocampal size, and by neuropathological studies reporting abnormal clustering of neurons [50,51].

Another possible mechanism by which DTNBP1 could modulate schizophrenia susceptibility, is by modulating prefrontal cortex (PFC) function. Reduced DTNBP1 expression has been reported in the dorsolateral PFC (DLPFC) in postmortem brain tissue of patients with schizophrenia [28]. Disturbed development of the DLPFC probably underlies cognitive dysfunction in schizophrenia and may contribute to schizophrenia pathogenesis [52]. Recently Kumamoto et al [30] reported that DTNBP1 also may be involved in the dopaminergic system, and may be capable of activating the midbrain dopaminergic system via indirect pathways [30]. Dopaminergic systems in the PFC and midbrain form a feedback mechanism and are modulated by genetic variation in dopamine clearance pathways; tuning of these systems is of critical importance for optimal cognitive functioning [53-56]. Thus, genetic variation in DTNBP1 may impact on dopaminergic and glutamatergic systems in the PFC and hippocampus, thereby disturbing memory and cognition and increasing schizophrenia susceptibility.

### Strengths and limitations

Strength of our study is the relatively young age of the patients (mean age was 21 years in our sample versus approximately 40 years in the sample of Burdick et al [32]), therefore potential confounders, e.g. disease duration or chronic antipsychotic treatment, are unlikely to influence IQ measurements. Limitations of our study are small sample size and the use of an ethnically mixed sample. To reduce the risk for population stratification, we (1) adjusted for ethnicity in the case-control analyses; (2) used unaffected siblings as controls; (3) matched the unrelated controls for ethnicity; and (4) used additional family-based analyses which confirmed results of the case-control analyses. Therefore, we think it is unlikely our results are due to admixture. Multiple testing effects could also allow the occurrence of type I errors. Because we expect the contribution of individual genes to the risk of complex genetic disorders to be small, and because the tests we performed are not completely independent (LD between SNPs is high and VIQ, PIQ, and FSIQ are highly correlated) we consider the Bonferroni method too strict to correct for type I error. However, we acknowledge that our results would not survive other – less stringent – methods to correct for multiple testing. Therefore our findings are preliminary and need to be replicated in a large and genetically homogeneous sample. Also, most of our included patients are male; therefore results may not generalize to female patients. Finally, although we found no correlation between chlorpromazine equivalents and FSIQ, the use of antipsychotic medication is a potential confounding factor in our study since all patients used antipsychotics whereas neither the siblings nor the unrelated controls used any psychotropic medication.

### Future directions

Future research will need to further investigate the complex genetic background of cognitive phenotypes in schizophrenia, including involvement of multiple susceptibility genes and gene-environmental interactions. Additionally, the use of combined techniques for measuring cognitive phenotypes, e.g. acquiring functional brain images during neuropsychological testing, may improve reliability and resolution of cognitive phenotypes in schizophrenia, thereby increasing power for molecular genetic studies.

## Conclusion

In this study, we report association between genetic variation in the DTNBP1 gene and IQ measures in patients with first-episode psychosis, unaffected siblings, and unrelated control subjects. There is growing evidence that genetic variation modulates cognition in schizophrenia; not only has genetic variation in DTNBP1 previously been associated with spatial working memory function and general intellectual function [32,33], but also variations in the DISC 1 gene have been related to structure and function of the hippocampus [57], and a functional variant in the NRG1 gene has been associated with impaired prefrontal function and decreased IQ [58]. Although preliminary, our results add to the growing body of research that genetic variation in DTNBP1 alters schizophrenia liability, possibly by affecting cognition.

## Abbreviations

ANOVA: Analysis of variance

DISC 1: Disrupted in schizophrenia 1 gene

DLPFC: Dorsolateral prefrontal cortex

DNA: Deoxyribonucleic acid

DSM-IV: Diagnostic and Statistical Manual of Mental Disorders, Fourth Edition

DTNBP1: Dystrobrevin-binding protein 1

ERP: Event-related potential

FEP: First-episode psychosis; first episode of schizophrenia or related psychotic disorder

FSIQ: Full scale intelligence quotient

GEE: Generalized estimating equations

MALDI-TOF MS: Matrix-assisted laser-desorption/ionization time-of-flight mass spectrometry

MINI: Mini International Neuropsychiatric Interview

NRG1: Neuregulin 1

PFC: Prefrontal cortex

PIQ: Performance intelligence quotient

QPDT: Quantitative pedigree disequilibrium test

SD: Standard deviation

SEM: Standard error of mean

SNP: Single nucleotide polymorphism

TDT: Transmission disequilibrium test

WAIS: Wechsler Adult Intelligence Test

VIQ: Verbal intelligence quotient

## Competing interests

The author(s) declare that they have no competing interests.

## Authors' contributions

JZ collected patients' material, carried out the molecular genetic studies, and drafted the manuscript, OdW collected clinical details and performed the neuropsychological tests, TvA participated in design and coordination of the study and helped to draft the manuscript, MT performed the statistical analyses, FB and DL conceived of the study and participated in its design. All authors read and approved the final manuscript.

## References

[B1] Heaton R, Paulsen JS, McAdams LA, Kuck J, Zisook S, Braff D, Harris J, Jeste DV (1994). Neuropsychological deficits in schizophrenics. Relationship to age, chronicity, and dementia. Arch Gen Psychiatry.

[B2] Green MF, Kern RS, Braff DL, Mintz J (2000). Neurocognitive deficits and functional outcome in schizophrenia: are we measuring the "right stuff"?. Schizophr Bull.

[B3] Kraepelin E (1971). Dementia praecox and paraphrenia.

[B4] Gold JM (2004). Cognitive deficits as treatment targets in schizophrenia. Schizophr Res.

[B5] Brekke JS, Hoe M, Long J, Green MF (2007). How Neurocognition and Social Cognition Influence Functional Change During Community-Based Psychosocial Rehabilitation for Individuals with Schizophrenia. Schizophr Bull.

[B6] Harrison PJ, Weinberger DR (2005). Schizophrenia genes, gene expression, and neuropathology: on the matter of their convergence. Mol Psychiatry.

[B7] Lewis DA, Moghaddam B (2006). Cognitive dysfunction in schizophrenia: convergence of gamma-aminobutyric acid and glutamate alterations. Arch Neurol.

[B8] Abi-Dargham A, Rodenhiser J, Printz D, Zea-Ponce Y, Gil R, Kegeles LS, Weiss R, Cooper TB, Mann JJ, Van Heertum RL, Gorman JM, Laruelle M (2000). Increased baseline occupancy of D2 receptors by dopamine in schizophrenia. Proc Natl Acad Sci U S A.

[B9] Norton N, Williams HJ, Owen MJ (2006). An update on the genetics of schizophrenia. Curr Opin Psychiatry.

[B10] Faraone SV, Seidman LJ, Kremen WS, Toomey R, Pepple JR, Tsuang MT (2000). Neuropsychologic functioning among the nonpsychotic relatives of schizophrenic patients: the effect of genetic loading. Biol Psychiatry.

[B11] Straub RE, MacLean CJ, O'Neill FA, Burke J, Murphy B, Duke F, Shinkwin R, Webb BT, Zhang J, Walsh D, . (1995). A potential vulnerability locus for schizophrenia on chromosome 6p24-22: evidence for genetic heterogeneity. Nat Genet.

[B12] Moises HW, Yang L, Kristbjarnarson H, Wiese C, Byerley W, Macciardi F, Arolt V, Blackwood D, Liu X, Sjogren B, . (1995). An international two-stage genome-wide search for schizophrenia susceptibility genes. Nat Genet.

[B13] Schwab SG, Hallmayer J, Albus M, Lerer B, Eckstein GN, Borrmann M, Segman RH, Hanses C, Freymann J, Yakir A, Trixler M, Falkai P, Rietschel M, Maier W, Wildenauer DB (2000). A genome-wide autosomal screen for schizophrenia susceptibility loci in 71 families with affected siblings: support for loci on chromosome 10p and 6. Mol Psychiatry.

[B14] Lewis CM, Levinson DF, Wise LH, DeLisi LE, Straub RE, Hovatta I, Williams NM, Schwab SG, Pulver AE, Faraone SV, Brzustowicz LM, Kaufmann CA, Garver DL, Gurling HM, Lindholm E, Coon H, Moises HW, Byerley W, Shaw SH, Mesen A, Sherrington R, O'Neill FA, Walsh D, Kendler KS, Ekelund J, Paunio T, Lonnqvist J, Peltonen L, O'donovan MC, Owen MJ, Wildenauer DB, Maier W, Nestadt G, Blouin JL, Antonarakis SE, Mowry BJ, Silverman JM, Crowe RR, Cloninger CR, Tsuang MT, Malaspina D, Harkavy-Friedman JM, Svrakic DM, Bassett AS, Holcomb J, Kalsi G, McQuillin A, Brynjolfson J, Sigmundsson T, Petursson H, Jazin E, Zoega T, Helgason T (2003). Genome scan meta-analysis of schizophrenia and bipolar disorder, part II: Schizophrenia. Am J Hum Genet.

[B15] Straub RE, Jiang Y, MacLean CJ, Ma Y, Webb BT, Myakishev MV, Harris-Kerr C, Wormley B, Sadek H, Kadambi B, Cesare AJ, Gibberman A, Wang X, O'Neill FA, Walsh D, Kendler KS (2002). Genetic variation in the 6p22.3 gene DTNBP1, the human ortholog of the mouse dysbindin gene, is associated with schizophrenia. Am J Hum Genet.

[B16] Morris DW, McGhee KA, Schwaiger S, Scully P, Quinn J, Meagher D, Waddington JL, Gill M, Corvin AP (2003). No evidence for association of the dysbindin gene [DTNBP1] with schizophrenia in an Irish population-based study. Schizophr Res.

[B17] Schwab SG, Knapp M, Mondabon S, Hallmayer J, Borrmann-Hassenbach M, Albus M, Lerer B, Rietschel M, Trixler M, Maier W, Wildenauer DB (2003). Support for association of schizophrenia with genetic variation in the 6p22.3 gene, dysbindin, in sib-pair families with linkage and in an additional sample of triad families. Am J Hum Genet.

[B18] Tang JX, Zhou J, Fan JB, Li XW, Shi YY, Gu NF, Feng GY, Xing YL, Shi JG, He L (2003). Family-based association study of DTNBP1 in 6p22.3 and schizophrenia. Mol Psychiatry.

[B19] Van Den Bogaert A, Schumacher J, Schulze TG, Otte AC, Ohlraun S, Kovalenko S, Becker T, Freudenberg J, Jonsson EG, Mattila-Evenden M, Sedvall GC, Czerski PM, Kapelski P, Hauser J, Maier W, Rietschel M, Propping P, Nothen MM, Cichon S (2003). The DTNBP1 (dysbindin) gene contributes to schizophrenia, depending on family history of the disease. Am J Hum Genet.

[B20] van den Oord EJ, Sullivan PF, Jiang Y, Walsh D, O'Neill FA, Kendler KS, Riley BP (2003). Identification of a high-risk haplotype for the dystrobrevin binding protein 1 (DTNBP1) gene in the Irish study of high-density schizophrenia families. Mol Psychiatry.

[B21] Funke B, Finn CT, Plocik AM, Lake S, DeRosse P, Kane JM, Kucherlapati R, Malhotra AK (2004). Association of the DTNBP1 locus with schizophrenia in a U.S. population. Am J Hum Genet.

[B22] Kirov G, Ivanov D, Williams NM, Preece A, Nikolov I, Milev R, Koleva S, Dimitrova A, Toncheva D, O'donovan MC, Owen MJ (2004). Strong evidence for association between the dystrobrevin binding protein 1 gene (DTNBP1) and schizophrenia in 488 parent-offspring trios from Bulgaria. Biol Psychiatry.

[B23] Numakawa T, Yagasaki Y, Ishimoto T, Okada T, Suzuki T, Iwata N, Ozaki N, Taguchi T, Tatsumi M, Kamijima K, Straub RE, Weinberger DR, Kunugi H, Hashimoto R (2004). Evidence of novel neuronal functions of dysbindin, a susceptibility gene for schizophrenia. Hum Mol Genet.

[B24] Williams NM, Preece A, Morris DW, Spurlock G, Bray NJ, Stephens M, Norton N, Williams H, Clement M, Dwyer S, Curran C, Wilkinson J, Moskvina V, Waddington JL, Gill M, Corvin AP, Zammit S, Kirov G, Owen MJ, O'Donovan MC (2004). Identification in 2 independent samples of a novel schizophrenia risk haplotype of the dystrobrevin binding protein gene (DTNBP1). Arch Gen Psychiatry.

[B25] Williams NM, O'donovan MC, Owen MJ (2005). Is the dysbindin gene (DTNBP1) a susceptibility gene for schizophrenia?. Schizophr Bull.

[B26] Posthuma D, Luciano M, Geus EJ, Wright MJ, Slagboom PE, Montgomery GW, Boomsma DI, Martin NG (2005). A genomewide scan for intelligence identifies quantitative trait loci on 2q and 6p. Am J Hum Genet.

[B27] Hallmayer JF, Kalaydjieva L, Badcock J, Dragovic M, Howell S, Michie PT, Rock D, Vile D, Williams R, Corder EH, Hollingsworth K, Jablensky A (2005). Genetic evidence for a distinct subtype of schizophrenia characterized by pervasive cognitive deficit. Am J Hum Genet.

[B28] Weickert CS, Straub RE, McClintock BW, Matsumoto M, Hashimoto R, Hyde TM, Herman MM, Weinberger DR, Kleinman JE (2004). Human dysbindin (DTNBP1) gene expression in normal brain and in schizophrenic prefrontal cortex and midbrain. Arch Gen Psychiatry.

[B29] Talbot K, Eidem WL, Tinsley CL, Benson MA, Thompson EW, Smith RJ, Hahn CG, Siegel SJ, Trojanowski JQ, Gur RE, Blake DJ, Arnold SE (2004). Dysbindin-1 is reduced in intrinsic, glutamatergic terminals of the hippocampal formation in schizophrenia. J Clin Invest.

[B30] Kumamoto N, Matsuzaki S, Inoue K, Hattori T, Shimizu S, Hashimoto R, Yamatodani A, Katayama T, Tohyama M (2006). Hyperactivation of midbrain dopaminergic system in schizophrenia could be attributed to the down-regulation of dysbindin. Biochem Biophys Res Commun.

[B31] Cropley VL, Fujita M, Innis RB, Nathan PJ (2006). Molecular imaging of the dopaminergic system and its association with human cognitive function. Biol Psychiatry.

[B32] Burdick KE, Lencz T, Funke B, Finn CT, Szeszko PR, Kane JM, Kucherlapati R, Malhotra AK (2006). Genetic variation in DTNBP1 influences general cognitive ability. Hum Mol Genet.

[B33] Donohoe G, Morris DW, Clarke S, McGhee KA, Schwaiger S, Nangle JM, Garavan H, Robertson IH, Gill M, Corvin A (2007). Variance in neurocognitive performance is associated with dysbindin-1 in schizophrenia: a preliminary study. Neuropsychologia.

[B34] Fanous AH, van den Oord EJ, Riley BP, Aggen SH, Neale MC, O'Neill FA, Walsh D, Kendler KS (2005). Relationship between a high-risk haplotype in the DTNBP1 (dysbindin) gene and clinical features of schizophrenia. Am J Psychiatry.

[B35] DeRosse P, Funke B, Burdick KE, Lencz T, Ekholm JM, Kane JM, Kucherlapati R, Malhotra AK (2006). Dysbindin genotype and negative symptoms in schizophrenia. Am J Psychiatry.

[B36] Pantelis C, Harvey CA, Plant G, Fossey E, Maruff P, Stuart GW, Brewer WJ, Nelson HE, Robbins TW, Barnes TR (2004). Relationship of behavioural and symptomatic syndromes in schizophrenia to spatial working memory and attentional set-shifting ability. Psychol Med.

[B37] Association AP (1994). Diagnostic and Statistical Manual of Mental Disorders.

[B38] Sheehan DV, Lecrubier Y, Sheehan KH, Amorim P, Janavs J, Weiller E, Hergueta T, Baker R, Dunbar GC (1998). The Mini-International Neuropsychiatric Interview (M.I.N.I.): the development and validation of a structured diagnostic psychiatric interview for DSM-IV and ICD-10. J Clin Psychiatry.

[B39] Sauer S, Gut IG (2002). Genotyping single-nucleotide polymorphisms by matrix-assisted laser-desorption/ionization time-of-flight mass spectrometry. J Chromatogr B Analyt Technol Biomed Life Sci.

[B40] Zeger SL, Liang KY (1986). Longitudinal data analysis for discrete and continuous outcomes. Biometrics.

[B41] Spielman RS, Ewens WJ (1996). The TDT and other family-based tests for linkage disequilibrium and association. Am J Hum Genet.

[B42] Dudbridge F (2003). Pedigree disequilibrium tests for multilocus haplotypes. Genet Epidemiol.

[B43] Fallgatter AJ, Herrmann MJ, Hohoff C, Ehlis AC, Jarczok TA, Freitag CM, Deckert J (2006). DTNBP1 (Dysbindin) Gene Variants Modulate Prefrontal Brain Function in Healthy Individuals. Neuropsychopharmacology.

[B44] Burdick KE, Goldberg TE, Funke B, Bates JA, Lencz T, Kucherlapati R, Malhotra AK (2007). DTNBP1 genotype influences cognitive decline in schizophrenia. Schizophr Res.

[B45] Pantelis C, Velakoulis D, McGorry PD, Wood SJ, Suckling J, Phillips LJ, Yung AR, Bullmore ET, Brewer W, Soulsby B, Desmond P, McGuire PK (2003). Neuroanatomical abnormalities before and after onset of psychosis: a cross-sectional and longitudinal MRI comparison. Lancet.

[B46] Bray NJ, Preece A, Williams NM, Moskvina V, Buckland PR, Owen MJ, O'donovan MC (2005). Haplotypes at the dystrobrevin binding protein 1 (DTNBP1) gene locus mediate risk for schizophrenia through reduced DTNBP1 expression. Hum Mol Genet.

[B47] Chiba S, Hashimoto R, Hattori S, Yohda M, Lipska B, Weinberger DR, Kunugi H (2006). Effect of antipsychotic drugs on DISC1 and dysbindin expression in mouse frontal cortex and hippocampus. J Neural Transm.

[B48] Gornick MC, Addington AM, Sporn A, Gogtay N, Greenstein D, Lenane M, Gochman P, Ordonez A, Balkissoon R, Vakkalanka R, Weinberger DR, Rapoport JL, Straub RE (2005). Dysbindin (DTNBP1, 6p22.3) is associated with childhood-onset psychosis and endophenotypes measured by the Premorbid Adjustment Scale (PAS). J Autism Dev Disord.

[B49] Nakazawa K, McHugh TJ, Wilson MA, Tonegawa S (2004). NMDA receptors, place cells and hippocampal spatial memory. Nat Rev Neurosci.

[B50] Harrison PJ (2004). The hippocampus in schizophrenia: a review of the neuropathological evidence and its pathophysiological implications. Psychopharmacology (Berl).

[B51] Geuze E, Vermetten E, Bremner JD (2005). MR-based in vivo hippocampal volumetrics: 2. Findings in neuropsychiatric disorders. Mol Psychiatry.

[B52] Lewis DA, Cruz D, Eggan S, Erickson S (2004). Postnatal development of prefrontal inhibitory circuits and the pathophysiology of cognitive dysfunction in schizophrenia. Ann N Y Acad Sci.

[B53] Meyer-Lindenberg A, Kohn PD, Kolachana B, Kippenhan S, McInerney-Leo A, Nussbaum R, Weinberger DR, Berman KF (2005). Midbrain dopamine and prefrontal function in humans: interaction and modulation by COMT genotype. Nat Neurosci.

[B54] Schott BH, Seidenbecher CI, Fenker DB, Lauer CJ, Bunzeck N, Bernstein HG, Tischmeyer W, Gundelfinger ED, Heinze HJ, Duzel E (2006). The dopaminergic midbrain participates in human episodic memory formation: evidence from genetic imaging. J Neurosci.

[B55] Winterer G, Weinberger DR (2004). Genes, dopamine and cortical signal-to-noise ratio in schizophrenia. Trends Neurosci.

[B56] Tunbridge EM, Harrison PJ, Weinberger DR (2006). Catechol-o-methyltransferase, cognition, and psychosis: Val158Met and beyond. Biol Psychiatry.

[B57] Callicott JH, Straub RE, Pezawas L, Egan MF, Mattay VS, Hariri AR, Verchinski BA, Meyer-Lindenberg A, Balkissoon R, Kolachana B, Goldberg TE, Weinberger DR (2005). Variation in DISC1 affects hippocampal structure and function and increases risk for schizophrenia. Proc Natl Acad Sci U S A.

[B58] Hall J, Whalley HC, Job DE, Baig BJ, McIntosh AM, Evans KL, Thomson PA, Porteous DJ, Cunningham-Owens DG, Johnstone EC, Lawrie SM (2006). A neuregulin 1 variant associated with abnormal cortical function and psychotic symptoms. Nat Neurosci.

